# Whole animal matrix-assisted laser desorption/ionization time-of-flight (MALDI-TOF) mass spectrometry of ticks – Are spectra of *Ixodes ricinus* nymphs influenced by environmental, spatial, and temporal factors?

**DOI:** 10.1371/journal.pone.0210590

**Published:** 2019-01-15

**Authors:** Axel Karger, Barbara Bettin, Joern M. Gethmann, Christine Klaus

**Affiliations:** 1 Institute of Molecular Virology and Cell Biology, Friedrich-Loeffler-Institut, Greifswald-Insel Riems, Germany; 2 Institute of Epidemiology, Friedrich-Loeffler-Institut, Greifswald-Insel Riems, Germany; 3 Institute of Bacterial Infections and Zoonoses, Friedrich-Loeffler-Institut, Jena, Germany; University of Maryland, College Park, UNITED STATES

## Abstract

In the recent years matrix-assisted laser desorption/ionization time-of-flight (MALDI-TOF) mass spectrometry (MS) has become a useful tool to characterize arthropod species and their different stages of development. It was reported for sand flies and mosquitoes at immature stages and also assumed for ticks that geographic location can have a subtle influence on MALDI-TOF mass spectra which allows the discrimination of animals with specific local variations of the MALDI-TOF MS phenotype. It is so far uncertain, however, if these mass-spectrometric differences are based on genetic variation or on spectral features which depend on environmental or temporal features. The aim of this study was to analyze the influence of the geographic location, environmental factors and the season of the year on the MALDI-TOF mass spectra of *Ixodes (I*.*) ricinus* nymphs and if spectral variation would allow to draw conclusions with respect to the tick’s provenience or conditions that influence the tick life cycle. Application of multivariate statistical models on spectra of ticks collected in different seasons and different habitats and locations within Germany showed that the impact of the location seemed to be small while season and habitat seemed to have stronger impact on the MALDI-TOF mass spectra. Possibilities and limitations of MALDI-TOF mass spectra to draw conclusions on the tick life cycle are discussed.

## Introduction

Over the past decades, the impact of arthropod-borne and especially tick-borne diseases on human and animal health has constantly increased and causing severe problems for the health care systems worldwide [1, 2]. Various tick species are vectors for a huge number of pathogens and can host and transmit bacterial, viral and protozoal agents [1, 3, 4]. In the recent years the usage of matrix-assisted laser desorption/ionization time-of-flight (MALDI-TOF) mass spectrometry (MS) has much expanded from its prime application, the identification of bacteria, and now includes many more disease-related applications like the taxonomic classification of arthropods and the characterization of immune cells [5]. MALDI-TOF MS is a robust and convenient alternative method to morphological or genetic determination of arthropods, for example when high numbers of ticks need to be classified, especially larvae and nymphs, damaged or engorged ticks [6, 7]. The method is quick, inexpensive, and highly reproducible. For field studies, the only prerequisite is a database of reference spectra from authentic samples as the identification process is based on pattern recognition algorithms rather than on the identification of any biomolecules. Although it has been shown for bacteria that some of the most prominent peaks represent ribosomal and stress proteins [8, 9] the identity of the biomolecules behind the mass peaks is usually not considered for MALDI-TOF MS based identification. For the classification of very closely related organisms multivariate statistical models are usually required as the spectral features that allow a group discrimination generally do not manifest in the presence or absence of single marker peaks but are hidden in complex quantitative mass signatures. The procedure is applicable to complete ticks as well as sampled haemolymph [10] or body parts of damaged or engorged ticks. The latter option could be especially useful for cases in which identification relies on ticks or tick parts removed from patients and contamination with blood from the host is problematic [6, 11]. In contrast to PCR-based techniques used for taxonomic purposes, like the sequence analysis of 16S rRNA, MALDI-TOF MS, which is a protein-based technique, has been shown to allow the differentiation of different developmental stages of tick specimens from the same species [6, 11].

It was reported for sand flies [12] and mosquitoes at immature stages [13] and was assumed for ticks from different locations in Ethiopia [14] that the geographic location can have a subtle influence on MALDI-TOF mass spectra which allows the discrimination of animals with specific local variations of the MALDI-TOF MS phenotype. The aim of this study was to analyze the influence of the geographic location and environmental factors like different habitats (meadow, forest) and the season of the year on the MALDI-TOF mass spectra of *Ixodes (I*.*) ricinus* nymphs and if such possible variation can be used to draw conclusions with respect to the tick’s provenience or environmental conditions that influence the tick life cycle. To this purpose, nymphs of *I*. *ricinus* were collected in different locations and habitats in Germany, at different times of the year (spring, summer, fall) and the influence of these parameters on the spectra were analyzed using multivariate statistical methods.

## Materials and methods

### Tick collection

Ticks were collected by flagging, immediately cooled on ice, morphologically differentiated and stored at -80°C for MALDI-TOF MS analysis. The following environmental parameters were documented: location, date and time of collection, habitat, air and soil temperature, and relative humidity. A minimum of ten nymphs of *I*. *ricinus* for each parameter set were then prepared and analyzed by MALDI-TOF MS (Table 1). Ticks were collected in six locations in the German federal states of Baden-Württemberg (BW), Bavaria (BY), Lower Saxony (LS), Mecklenburg-West Pomerania (MV), North Rhine-Westphalia (NRW), Saarland (SL) (GPS-data in Table 1). The maximum distance between two single collection sites was 656 km as the crow flies (Groß Quassow, MV, and Hausach, BW). Nymphs were collected in two habitats (forest and meadow) at each location around the year. After a preliminary analysis indicating that the influence of different parameters on the spectra was generally only small, samples were selected for which we expected a measurable effect on the spectra. These included locations separated by large distances (BW, BY and MV) and the two habitats sampled in March, June and October 2009 and 2010.

**Table 1:** Characteristics of collection sites and sample numbers.

ID	federal state	region	habitat	season	sample number	GPS coordinates	
						N	E
1	Baden-Württemberg	Hausach	forest	Su	29	48°16’04.1	08°10’03.0
2	Bavaria	Loderhof	forest	Su	10	48°44’16.6”	13°20’48.0”
3	Lower Saxony	Holtum-Geest	forest	NA	NA	53°05’36.6”	09°22’26.9”
4	Mecklenburg-West Pomerania	Groß-Quassow	forest	Sp	10	53°18’39.0”	13°00’05.6”
5				Su	30		
6	Mecklenburg-West Pomerania	Voßwinkel	meadow	Sp	10	53°18’53.6”	13°02’14.4”
7				Fa	10		
8	North Rhine-Westphalia	Büren	forest	NA	NA	51°33’18.9”	08°27’07.3”
9	Saarland	Glashütter Weiher	forest	NA	NA	49°17’52.4	07°10’47.2”

Fa = fall, Sp = spring, Su = summer, NA = data not available

### Sample preparation for MS analysis

Single nymphs were homogenized in 300μl Earle’s MEM (Biochrom AG, Berlin, Germany) using a ball mill (Retsch GmbH, Haan, Germany, 30Hz, 2min) with three stainless steel balls (3mm, Isometall Handelsgesellschaft Schmidt & Co., Pleidelsheim, Germany). For the preparation of the MALDI target spot, a standard protocol for bacterial samples [15] was adapted. Ethanol was added to the homogenate to a final concentration of 67% (vol/vol). The precipitate was collected by centrifugation (14,000 rpm, 5 min), washed once with 500 μl 70% ethanol, and resuspended in 20 to 50μl 70% formic acid. After addition of the same volume acetonitrile, unsolved material was removed by centrifugation (14,000 rpm, 5 min) and 1.5 μl of the extract was spotted onto a MALDI target plate. After drying, the spot was overlaid with 2μl of a α-cyano-4-hydroxycinnamic acid (HCCA) matrix solution (HCCA saturated in 50% ACN/2.5% trifluoroacetic acid). Every sample was spotted sixfold on a MALDI steel target and from every spot 4 spectra were acquired for the generation of main spectra projection (MSP) as suggested by the manufacturer of the Biotyper and ClinProTools software (Bruker Daltonics, Bremen, Germany). Single spectra were inspected visually and spectra of low quality were excluded, leaving a minimum of 20 single spectra per sample for the calculation of a MSP.

### Statistical analysis

MSPs were calculated from the single spectra using Biotyper software (version 2.0, Bruker Daltonics, Bremen, Germany) following the guidelines of the manufacturer. For the preprocessing and the MSP generation the default parameter sets were applied. Score oriented dendrograms were constructed from the MSP using the spectra correlation as distance measure and the Ward linkage algorithm. For the evaluation with ClinProTools software (version 2.2, Bruker Daltonics, Bremen, Germany) the spectra were loaded and preprocessed using the default parameters. For the ‘spectra preparation’ option, a minimal resolution of 800 was chosen, the base line was calculated with the Top Hat algorithm and spectra were smoothed with 10 cycles of the Savitsky/Golay algorithm with a width of 2 m/z. Peaks were calculated on basis of the average spectra with minimal s/n ratios of 2.

Statistical models were calculated for pairs of sampling conditions with ClinProTools software using all 4 available algorithms, the genetic algorithm (GA), support vector machine (SVM), supervised neural network (SNN), and the quick classifier (QC). The suggested standard parameters were applied for GA (maximum of 10 peaks, maximum of 3 generations, k-nearest neighbor (KNN) classification with 3 neighbors), SVM (automatic detection of 1–25 peaks, KNN with 3 neighbors), SNN (automatic peak detection 1–15 peaks), and QC (automatic peak detection 1–25 peaks, p-value T-test/ANOVA). The cross validation was performed using the random mode with 50% leave out and 10 iterations.

### Permissions and ethical statement

In Germany legal guidelines allow free access to forest for everybody unless there are special reasons for restrictions such as status as national park or other protected areas. The study was carried out at locations for which no permission for entering and collecting of ticks was required. So all locations were in areas without a special protection status and permission for entering was not required (see above). The study did not involve endangered or protected species. No vertebrates were included in the study.

## Results

**Fig 1:**
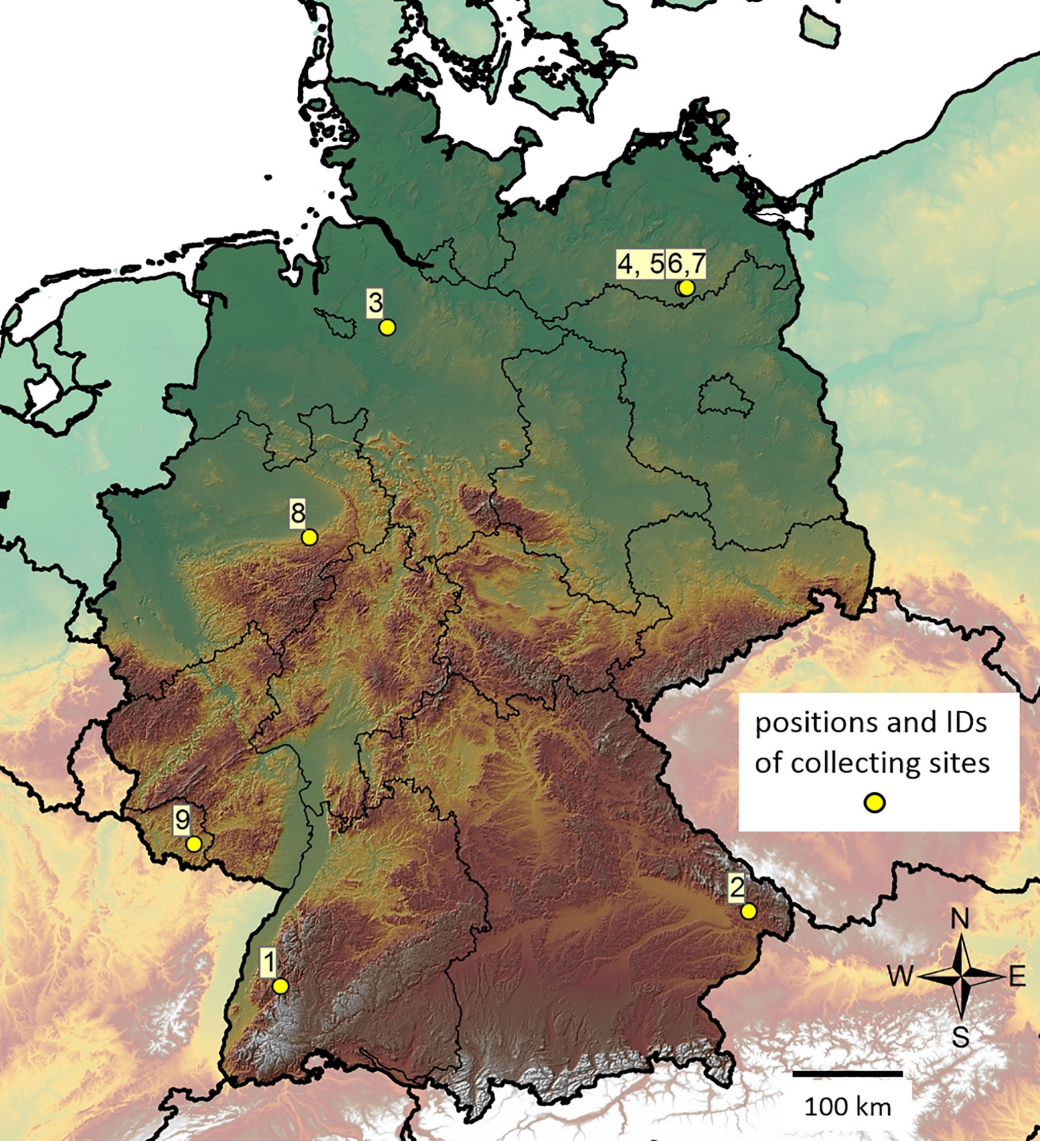
Map of *I*. *ricinus* collection sites in Germany. Numbers refer to sample IDs in Table 1, first column. On this scale, sites representing groups 4 and 5, and 6 and 7, respectively, are not resolved, as they are only 2.4 km apart.

**Fig 2:**
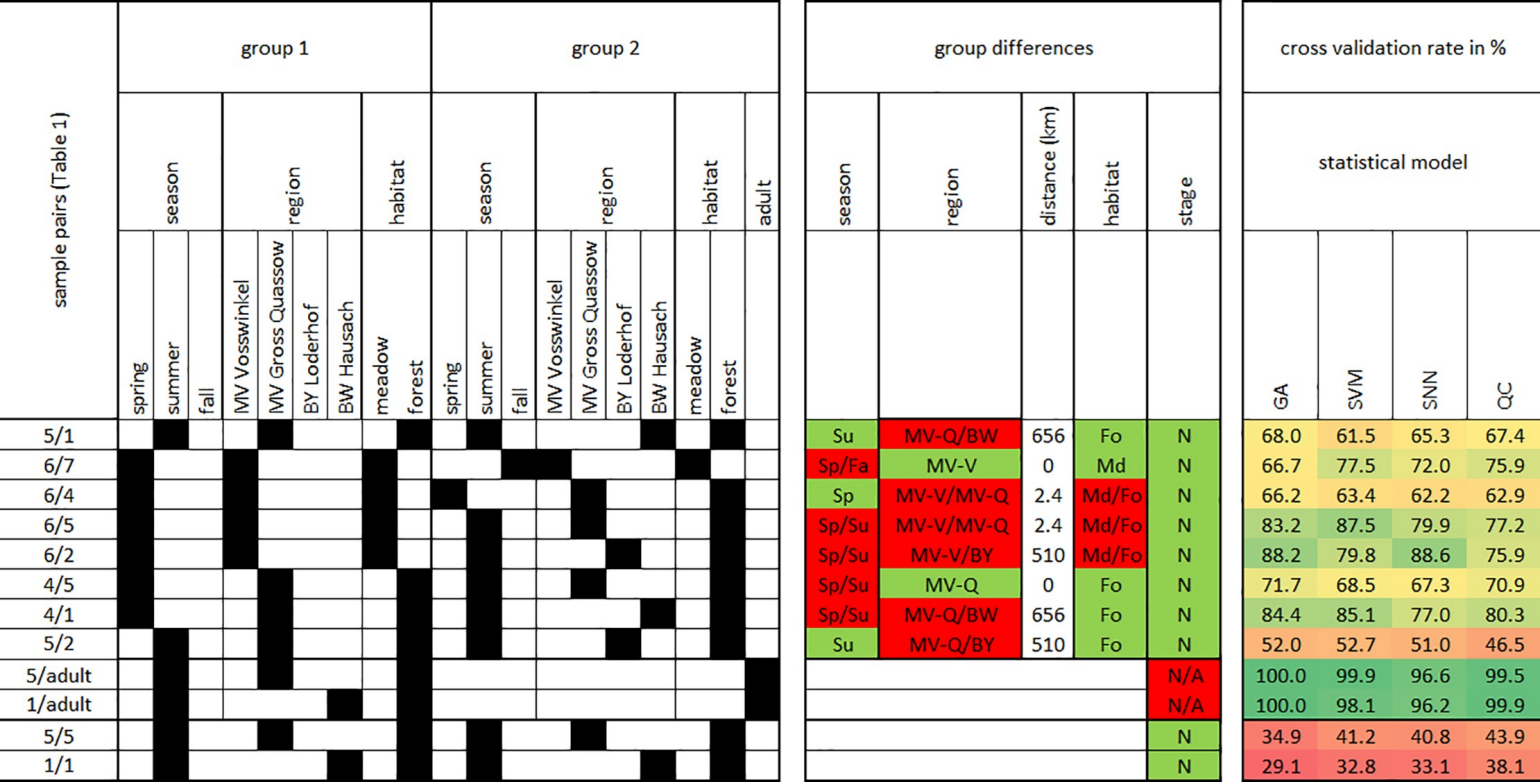
Multivariate models for ticks collected under different environmental, spatial, and temporal conditions. Specimens collected under different conditions were analyzed pairwise by calculation of statistical models. In the left panel, the column ‘sample pairs’ indicates the two groups which were analyzed in every row. Numbers refer to the sample IDs given in Table 1. Environmental parameters are given in the columns ‘season’, ‘region’ and ‘habitat’ for both groups as black boxes. In the central panel (‘group differences’), differing and concordant environmental parameters are highlighted in red and green color, respectively, and the distance between the two collection sites is given in km in the ‘distance’ column. The following abbreviations were used: Fa = fall, Sp = spring, Su = summer, Fo = forest, Md = meadow, N = nymphs, A = adult. In the right panel, the rates of correct cross validation with ClinProTools software is given in % for every pair and for the following algorithms: genetic algorithm (GA), support vector machine (SVM), supervised neural network (SNN), and the quick classifier (QC). Details of the statistical models are given as Supporting information in files S1 Table and S2 Table.

Spectra from *Ixodes ricinus* nymphs collected under the conditions described in Table 1 were processed with Biotyper and ClinProTools software for statistical analysis. The aim of this study was to identify the influence of environmental factors on tick nymph spectra. Samples originated from seven different locations within Germany (Fig 1) and comprised a minimum of 10 individual nymphs for every set of environmental parameters (Table 1). As preliminary studies had shown that variation of the spectra was generally very small, four locations were selected for which we expected a measurable effect of on the spectra (see Fig 2). Different habitats were sampled in different seasons of the same year. The resulting spectra were then evaluated pairwise using the ClinProTools software (Bruker). This software has been designed for the evaluation of mass spectra by multivariate statistics. Spectra representing samples subject to different experimental conditions, in our study combinations of environmental factors, are treated as ‘classes’ for which statistical models are calculated. The models can then be cross-validated using different schemes and unknown samples classified based on the validated models. Quantitative evaluation of the peak intensities can be used to identify masses that are discriminative for the classes and may represent markers for the respective experimental conditions. We have used all four available algorithms (see Methods section for details) for the calculation of multivariate models for the classes and have chosen a resampling scheme with 10 iterations of 50% leave out sampling for the cross-validation of the models. Spectra from adult *I*. *ricinus* served as a positive control for which we expected cross validation rates close to 100%, as it has been shown before that *I*. *ricinus* nymphs and adult animals can be well separated on basis of MALDI-TOF mass spectra [11]. We have not attempted to improve the performance of the statistical models by manual selection of mass peaks to avoid over-fitting but rather applied the default parameters offered by the ClinProTools software. Original spectra, parts of the graphic output from ClinproTools software, the details of the calculated models and the peak statistics are supplied as supplemental files S1 Fig, S1 Table, and S2 Table, respectively. The resulting percentage of correctly identified samples in the cross validation process served as a measure for the impact of the sampling parameters (location, habitat, season) on the spectra. To assess any over-fitting by the statistical models with the given parameters, 2 x 10 samples of the two larger groups from BW and MV (lines 1 and 5 in Table 1, respectively) were randomly selected and statistical models were calculated on basis of the random samples with identical parameter sets (last 2 lines of Fig 2). With values between 34 and 45% these random sample groups showed markedly lower cross validation rates than the statistical models for the groups with differing parameters where the lowest cross validation rate was 46.5%. As shown in Fig 2, the impact of the location within Germany alone (Fig 2, pairs 5/1 and 5/2) seemed to be small, as the successful cross validation ranged among the lower values and were the smallest observed for all four algorithms when applied to pair 5/2 (46.5 to 52.7%). Season alone seemed to have a stronger impact (pairs 6/7 and 4/5). Interestingly, pairwise comparison with samples differing in more than one parameter usually had better cross validation results than comparisons of pairs with only one differing parameter. Thus, variation introduced by location, season, or habitat seemed to add up allowing the fit of statistical models with, in some cases, excellent cross validation results up to over 88% (Fig 2, pair 6/2 differing in region, season and habitat).

**Fig 3:**
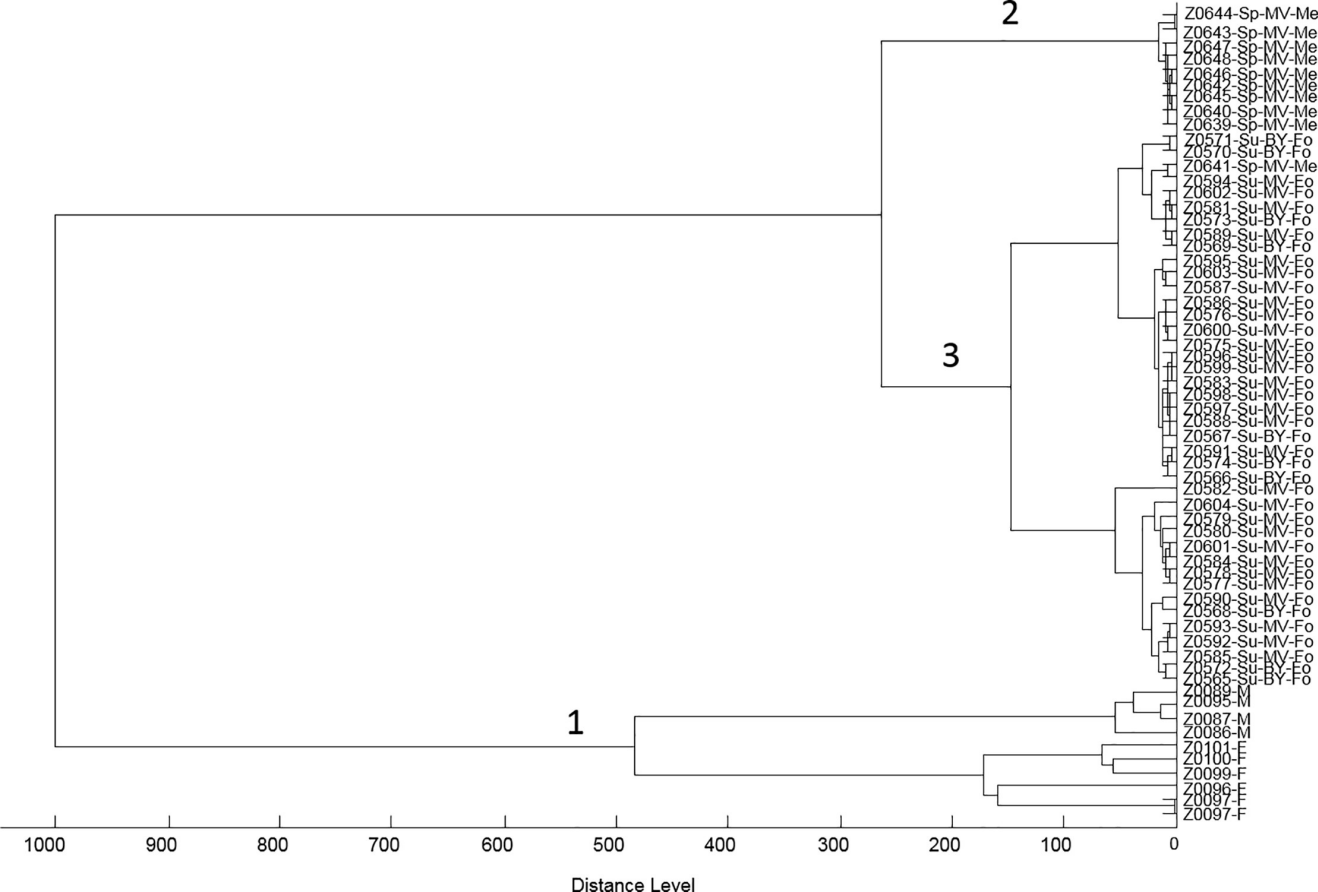
Spectrum-based dendrogram. Three groups of nymphs were included in the dendrogram which were collected during spring in a meadow in Mecklenburg-West Pomerania (Sp-MV-Me) or during summer in a forest in Mecklenburg-West Pomerania (Su-MV-Fo) or Bavaria (Su-BY-Fo). Adult *I*. *ricinus* spectra from female (F) and male (M) animals were added (cluster 1) and appeared clearly separated from the nymphs as expected. Nymph spectra separated in two main clusters primarily according to a combination of habitat and season (clusters 2 and 3). Spectra representing samples with differing provenience only (MV or BY, both collected in summer and in a forest) formed a joint group (3) indicating that the geographic location alone does not represent a strong determinant of the spectral phenotype.

The dendrogram in Fig 3 confirms these results. Three groups of nymphs were included in the dendrogram which were collected during spring in a meadow in Mecklenburg-West Pomerania or during summer in a forest in Mecklenburg-West Pomerania or Bavaria. While the adult animals were well separated from the nymphs as expected, the main clusters formed between samples differing in two parameters (habitat and season). The geographic location alone did not seem to impact strongly on the spectral phenotype as samples from different locations (MV and BY) as the only difference formed a mixed cluster.

## Discussion

We have observed in our study that spectra of ticks collected in different regions, habitats and in different seasons of the same year varied, and it was possible to calculate multivariate statistical model with high cross validation rates (46.5–88.6%, Fig 2) which all exceeded the cross validation rates observed for models that were calculated for resampled representatives of the same group (below 44%, Fig 2). Samples differing in more than one parameter tended to result in higher correct cross validation rates, indicating that the impacts of environmental and temporal factors on the spectra were additive. It is noteworthy that the best cross validation results were obtained for sample pairs differing, among other parameters, in the season (Fig 2, pairs 6/5, 6/2, 4/1) indicating that seasonal factors like humidity and temperature may have a stronger impact on the spectrum characteristics than region or habitat. Likely, the seasonal changes in the spectra may reflect gradual maturation of the tick nymphs over the season. This would corroborate the finding that developmental stages are strong determinants for the spectra (Fig 2, [11]). In contrast, the influence of the habitat and region on the spectra seemed to be less pronounced, arguing against the evolution of genetic variants in certain geographic regions.

*I*. *ricinus* ticks are well adapted to a wide range of environmental and other factors like habitat, season, potential host species and, with some limitations, temperature and humidity. We show here that environmental factors may have an additive influence on the characteristics of the MALDI-TOF mass spectra of ticks. Only in cases of temperature and humidity near the physiological constraints spectra might differ as a phenotypical result of stress situation. We would like to point out that environmental factors like temperature, humidity, and altitude may impact the spectra of tick specimens to a degree which may blur or even impede the detection of subtle genetic variations by MALDI-TOF MS. Therefore, it seems to be of great importance for the study of genetic variations in arthropods by MALDI-TOF MS to use samples which are matched for the environmental factors which also influence the spectral characteristics and may confound the statistical analysis. Even more variation might conceivably be introduced directly or indirectly by other factors like the identity of the host of the last blood meal which can in turn impact the tick microbiome [16] and thus, the overall composition of the mass spectrometric sample. Dependence of the spectra on environmental factors could be interpreted as limitation for the use of MALDI-TOF MS as convenient surrogate for a genetic analysis. On the other hand, MALDI-TOF MS indeed is a phenotypic analysis targeting proteins rather than nucleic acids and therefore may be used to disclose interactions of the tick with its environment which are obscure for genetic analysis. For *Amblyomma variegatum* (Fabricius) ticks such a genetic segregation into distinct East and West African groups has been documented by Beati et al. [17] which may also lead to different regional MALDI-TOF MS phenotypes. Indeed, regional MALDI-TOF variation has been described for sandflies and mosquitoes [12, 13] and is also assumed for ticks from several regions of Ethiopia [14]. But it also seems conceivable that ticks living at different places and habitats may respond to the given environmental conditions by phenotypic rather than genetic adaption which may impact MALDI-TOF mass spectra and cause the minor spectral variations that we and others have observed. The collection sites of the present study were less than 660 km apart, and we have observed slight spectral differences between samples collected at different times of the year but at the same location and habitat. The close regional and temporal proximity of the samples argue against the development of genetic variants but are in favor of a flexible phenotypical reaction of ticks to environmental conditions which impact the spectral phenotype. Thus, MALDI-TOF MS and genetic analysis should be seen as complementary rather than competitive approaches for the analysis of ticks.

## Supporting information

S1 Fig Spectra and evaluation with ClinProTools. Sheets 1–12 show details of the evaluation with ClinProTools for the 12 pairs of samples from Fig 2. In each of these sheets, the overlays of the average spectra (upper left), the magnifications of the regions with the two peaks showing the largest difference in intensities (right) and the dot plot of the intensities of the same two peaks for the centroid spectra of every group member (lower left) are shown. Overlays of original spectra copied from Flexanalysis software (Bruker) were added (whole spectra and 5 magnified m/z regions) for pairs of samples which are considered very similar (sheets 13 and 14, spectra resampled from group 1, line 12 in Fig 2), very distant (sheets 15 and 16, spectra from nymphs and adult animals, line 10 in Fig 2), and a pair of nymph samples with high cross validation rates (sheets 17 and 18, line 5 in Fig 2).
(PPTX)

S1 Table Statistic evaluation of peak intensities. A detailed description of the presented data is included in the datasheet ‘legend’.
(XLSX)

S2 Table Statistical models calculated with ClinProTools. Detailed presentation of the model parameters in Fig 2 calculated with ClinProTools.
(XLSX)
