# Supplementary material for: Whole animal matrix-assisted laser desorption/ionization time-of-flight (MALDI-TOF) mass spectrometry of ticks – Are spectra of Ixodes ricinus nymphs influenced by environmental, spatial, and temporal factors?
Source: PLoS One. 2019 Jan 15;14(1):e0210590. doi: 10.1371/journal.pone.0210590 (PMC6333373; doi:10.1371/journal.pone.0210590)
Supplement: S1 Fig — Sheets 1–12 show details of the evaluation with ClinProTools for the 12 pairs of samples from Fig 2. In each of these sheets, the overlays of the average spectra (upper left), the magnifications of the regions with the two peaks showing the largest difference in intensities (right) and the dot plot of the intensities of the same two peaks for the centroid spectra of every group member (lower left) are shown. Overlays of original spectra copied from Flexanalysis software (Bruker) were added (whole spectra and 5 magnified m/z regions) for pairs of samples which are considered very similar (sheets 13 and 14, spectra resampled from group 1, line 12 in Fig 2), very distant (sheets 15 and 16, spectra from nymphs and adult animals, line 10 in Fig 2), and a pair of nymph samples with high cross validation rates (sheets 17 and 18, line 5 in Fig 2). (PPTX) [file pone.0210590.s001.pptx]

## Slide 1
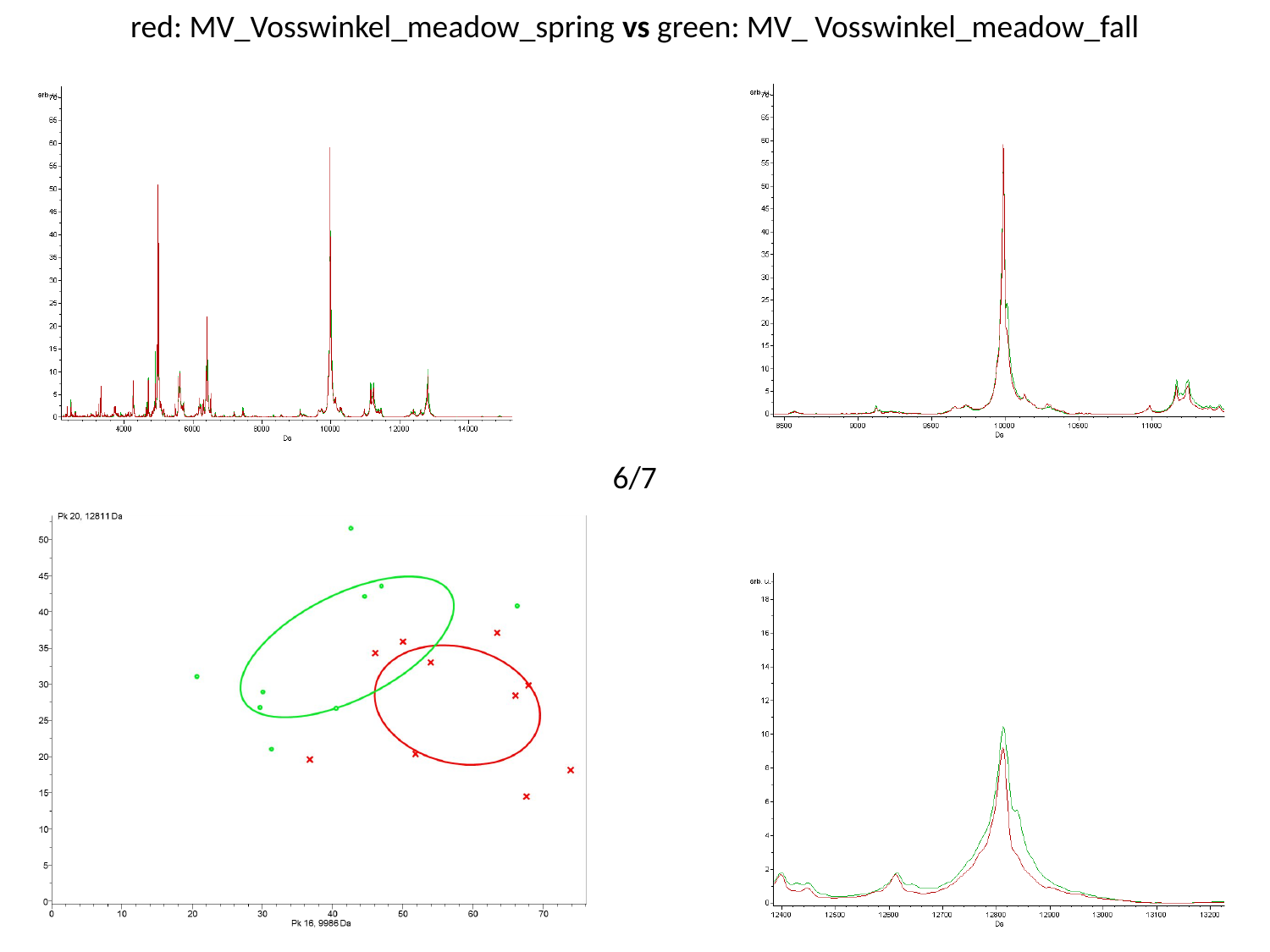

red: MV_Vosswinkel_meadow_spring vs green: MV_ Vosswinkel_meadow_fall
6/7

## Slide 2
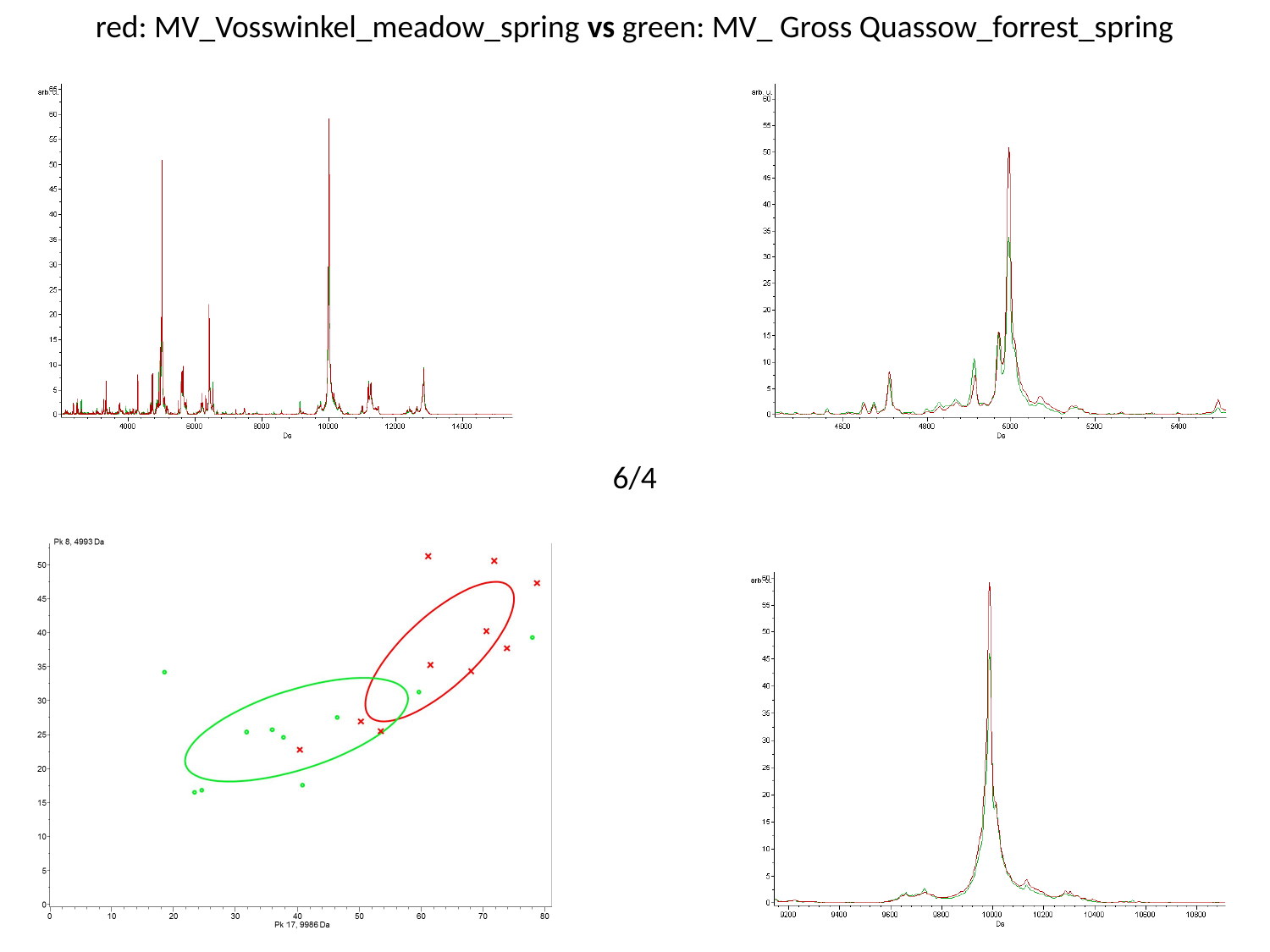

red: MV_Vosswinkel_meadow_spring vs green: MV_ Gross Quassow_forrest_spring
6/4

## Slide 3
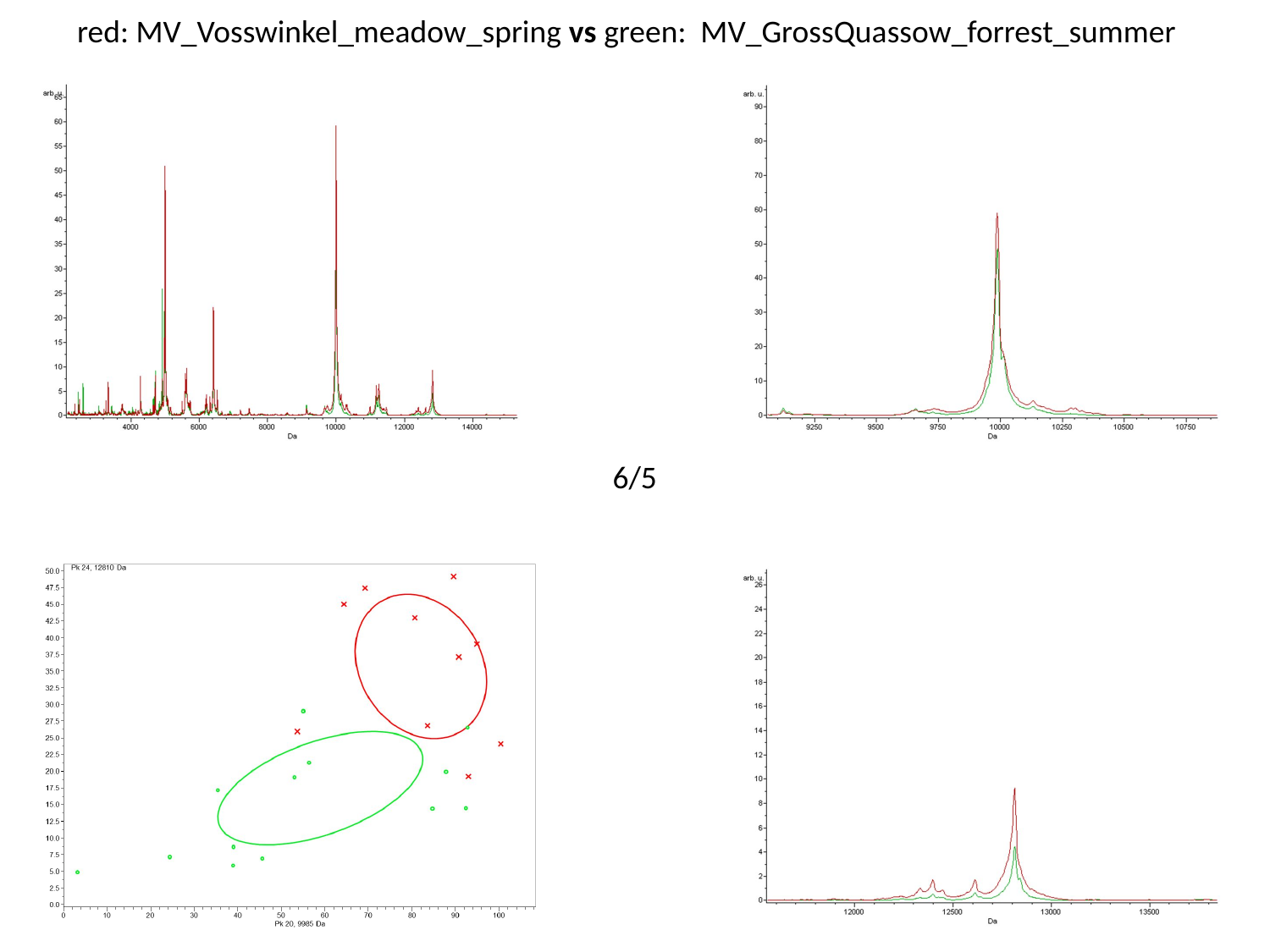

red: MV_Vosswinkel_meadow_spring vs green: MV_GrossQuassow_forrest_summer
6/5

## Slide 4
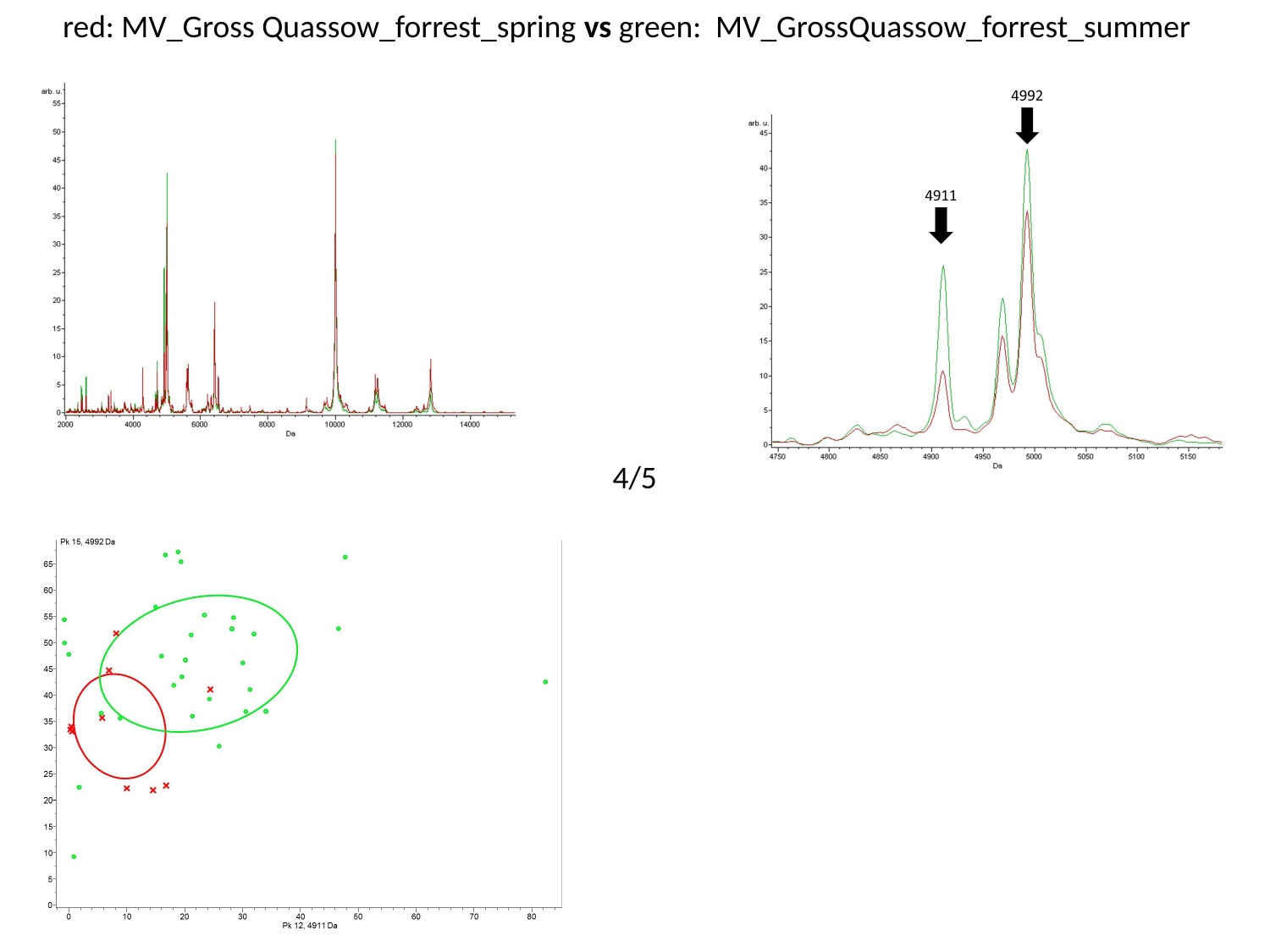

red: MV_Gross Quassow_forrest_spring vs green: MV_GrossQuassow_forrest_summer
4/5

## Slide 5
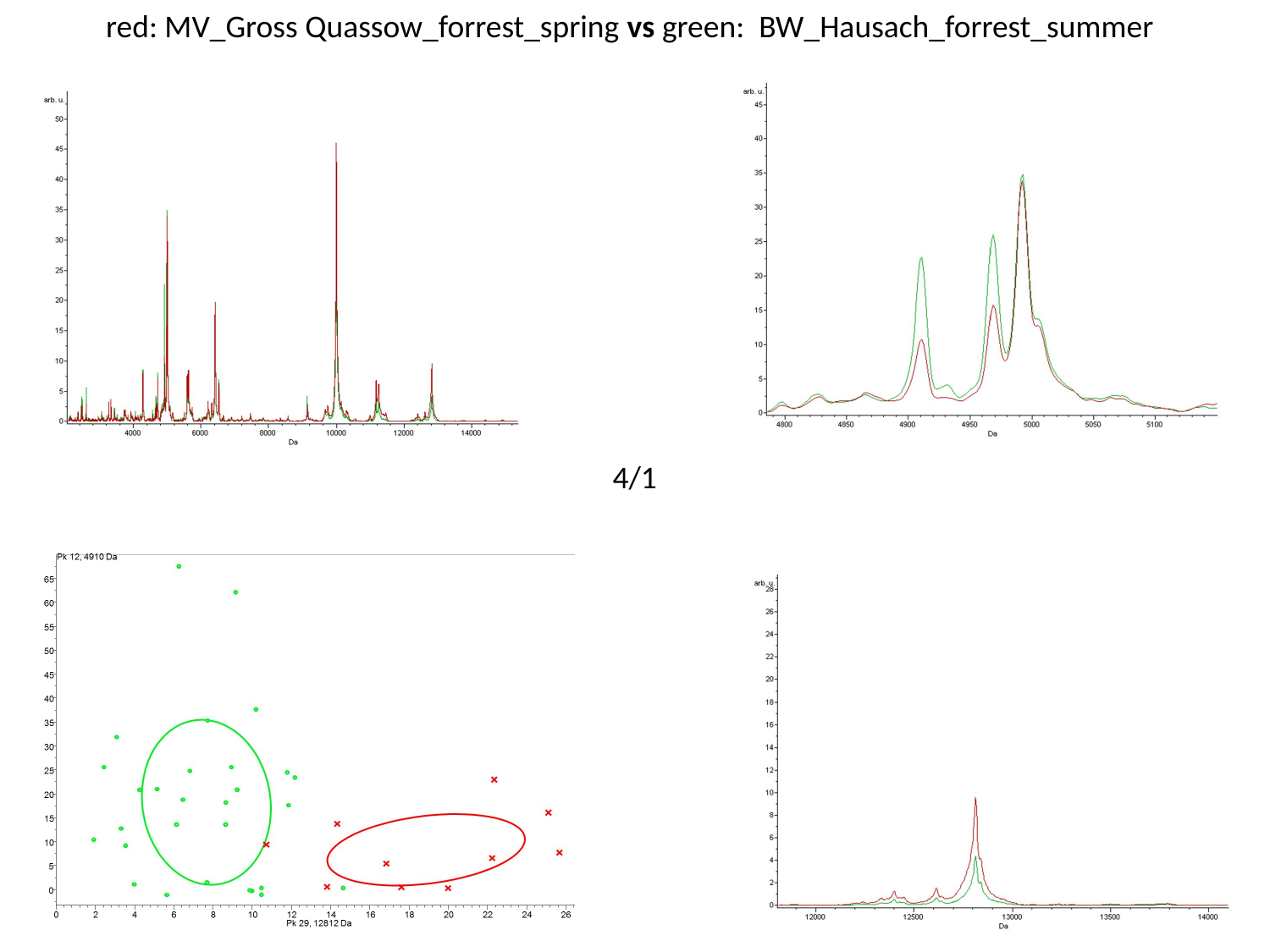

red: MV_Gross Quassow_forrest_spring vs green: BW_Hausach_forrest_summer
4/1

## Slide 6
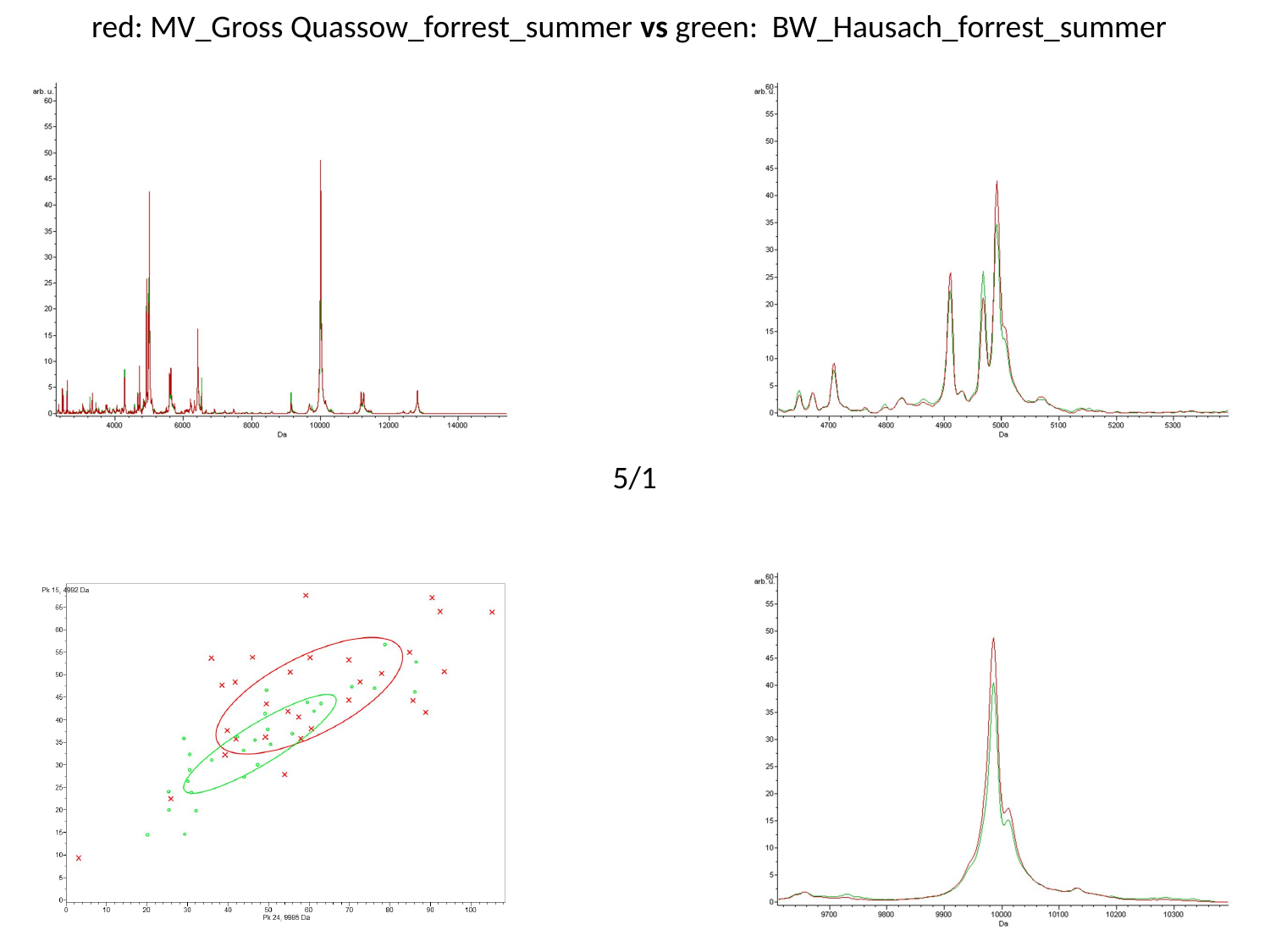

red: MV_Gross Quassow_forrest_summer vs green: BW_Hausach_forrest_summer
5/1

## Slide 7
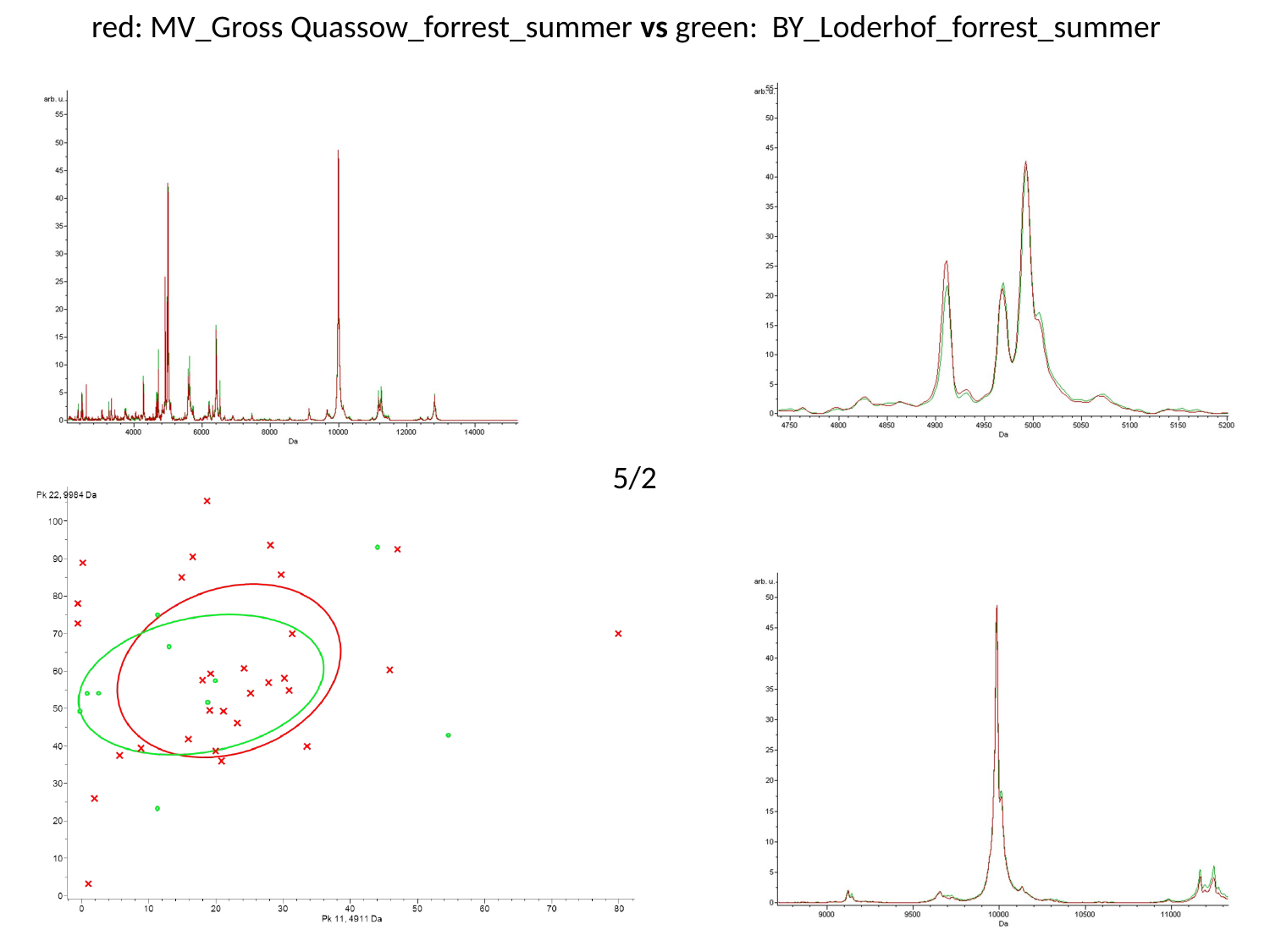

red: MV_Gross Quassow_forrest_summer vs green: BY_Loderhof_forrest_summer
5/2

## Slide 8
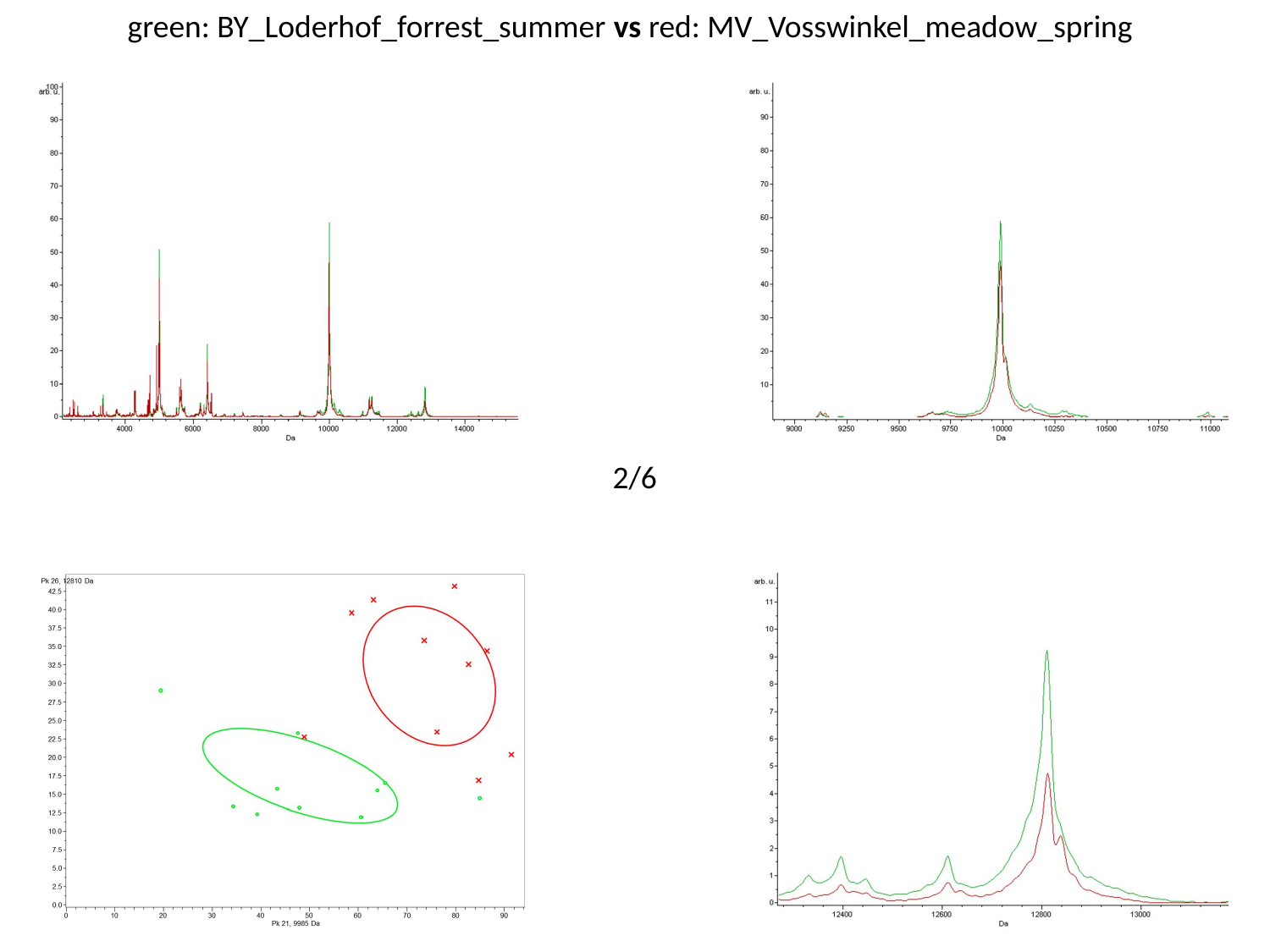

green: BY_Loderhof_forrest_summer vs red: MV_Vosswinkel_meadow_spring
2/6

## Slide 9
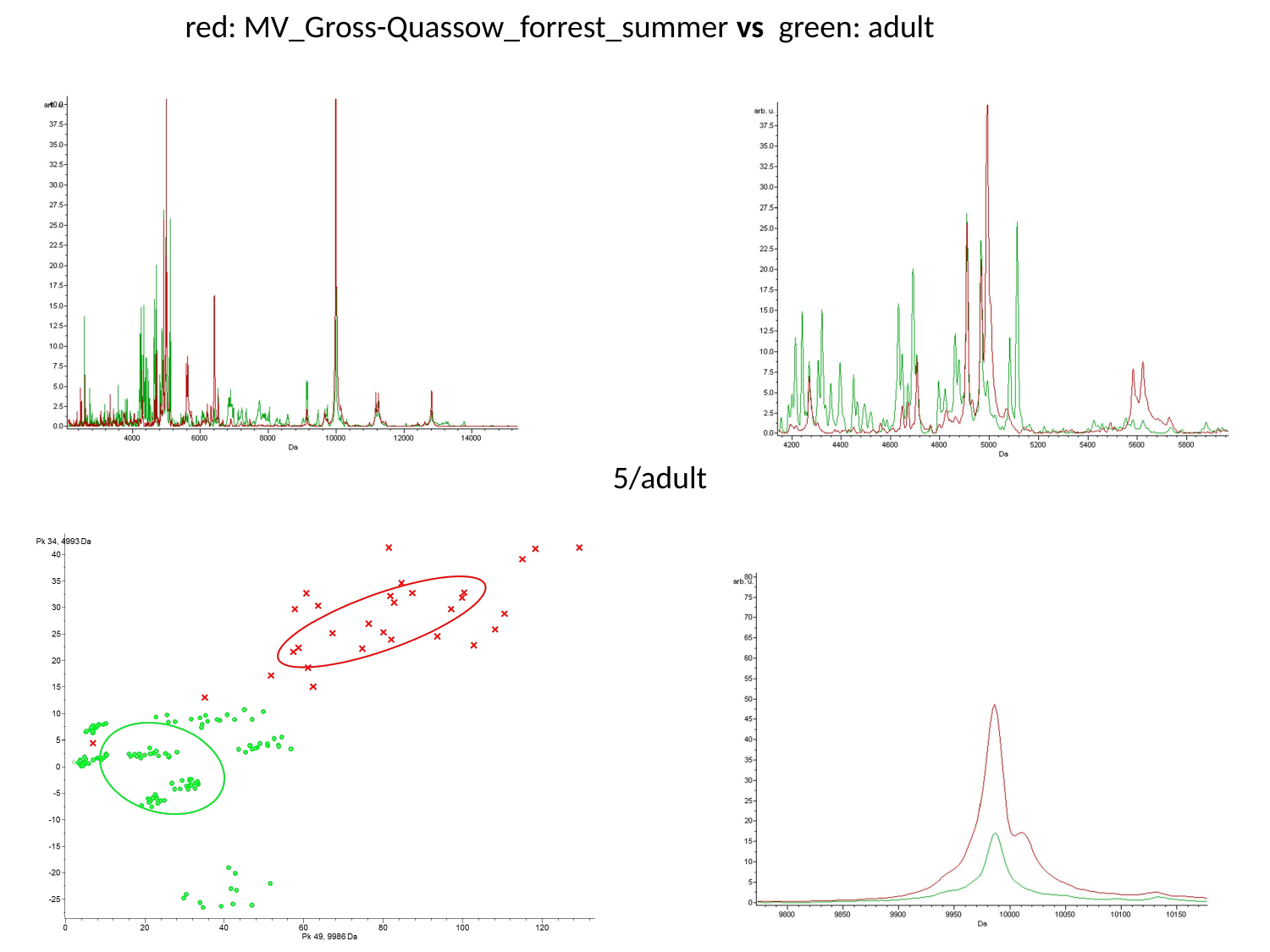

red: MV_Gross-Quassow_forrest_summer vs green: adult
5/adult

## Slide 10
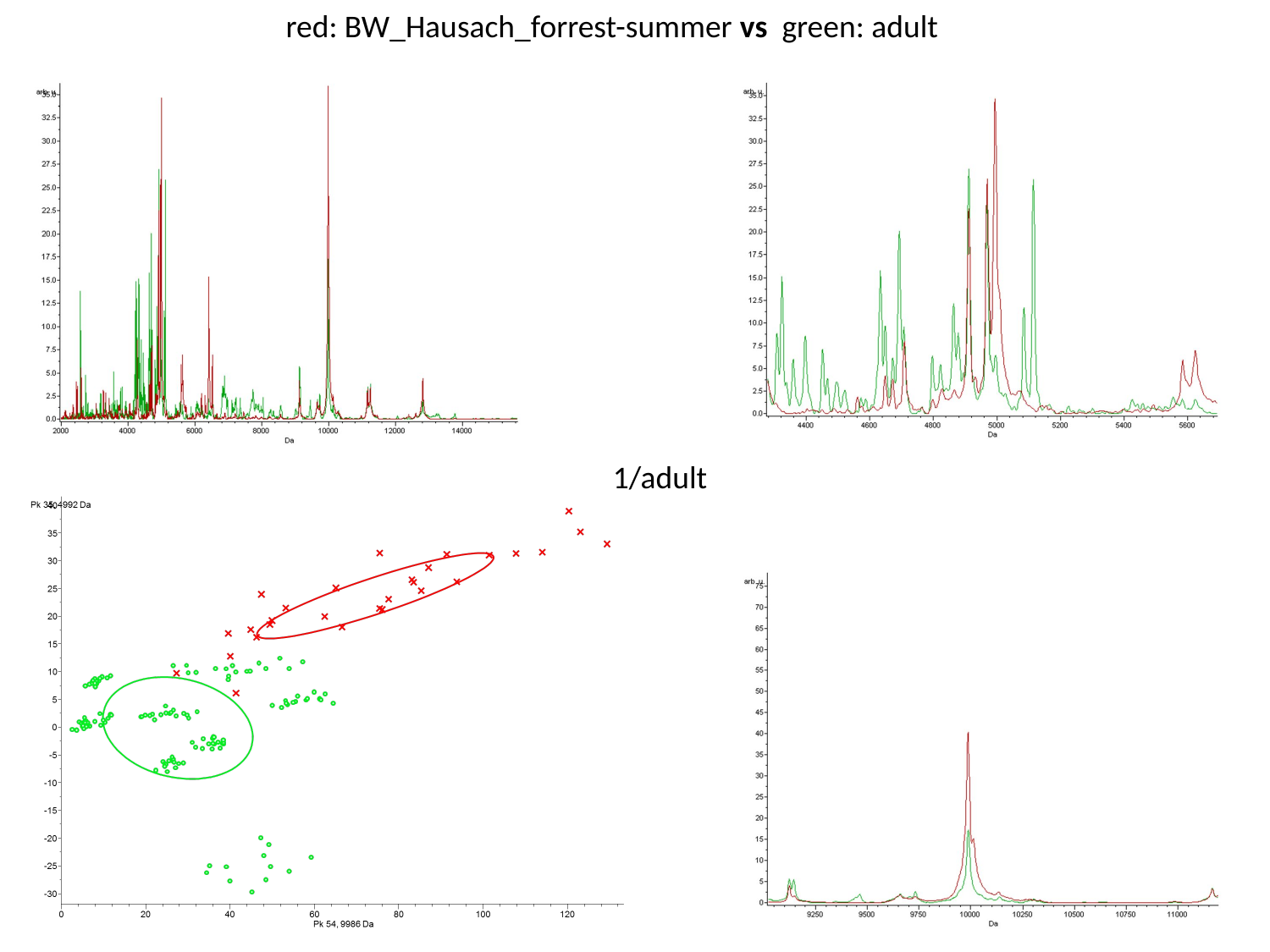

red: BW_Hausach_forrest-summer vs green: adult
1/adult

## Slide 11
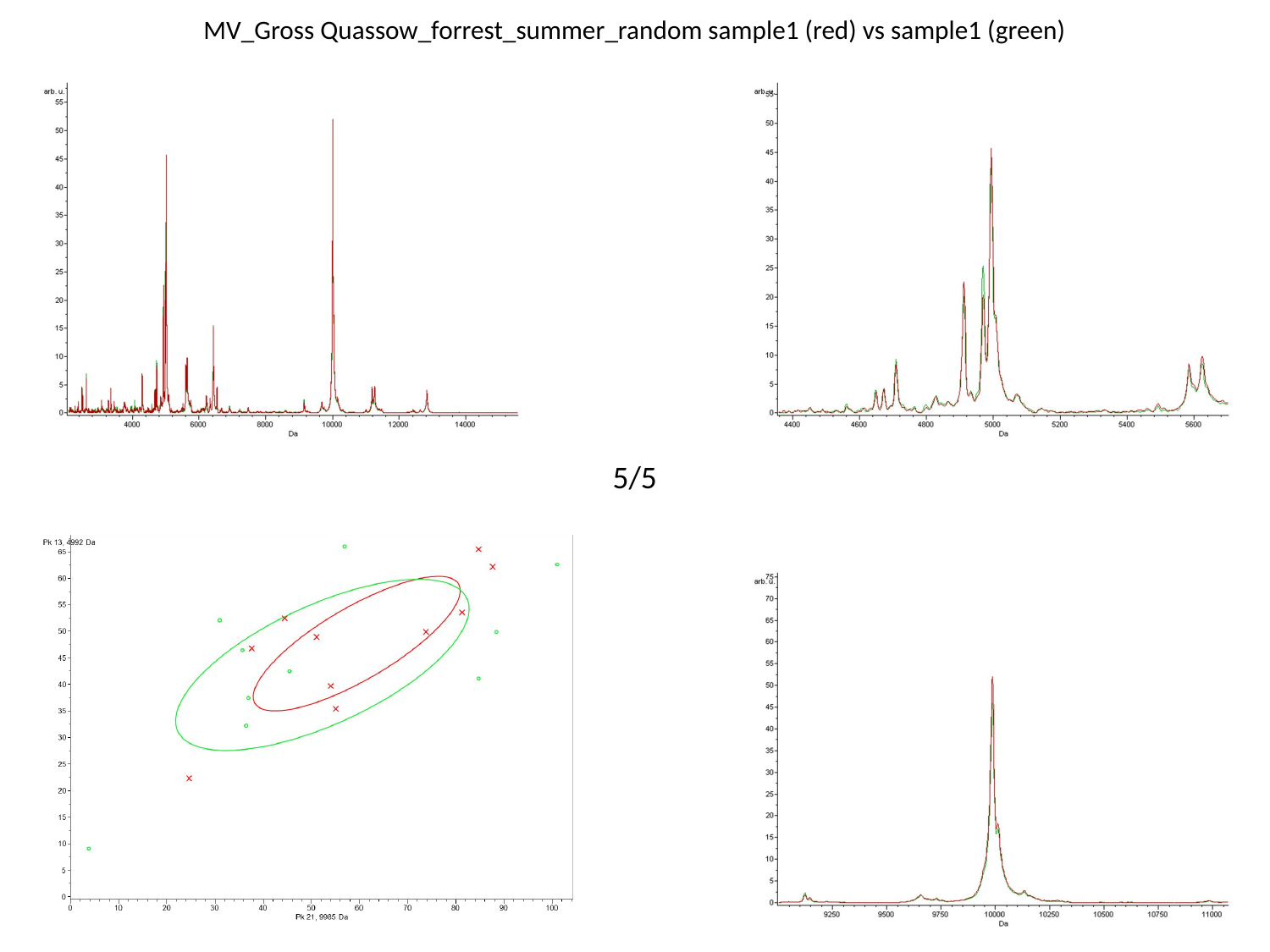

MV_Gross Quassow_forrest_summer_random sample1 (red) vs sample1 (green)
5/5

## Slide 12
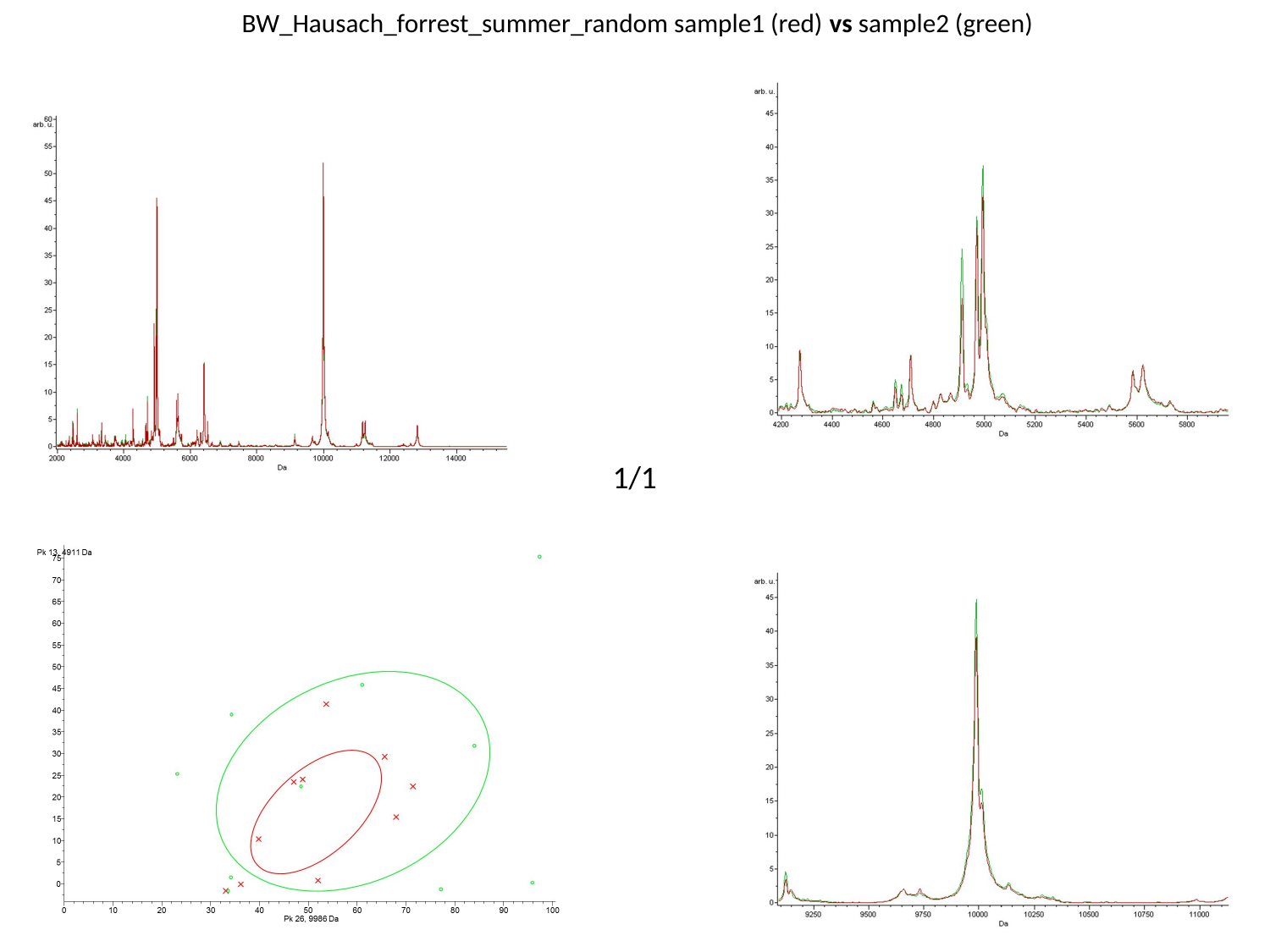

BW_Hausach_forrest_summer_random sample1 (red) vs sample2 (green)
1/1

## Slide 13
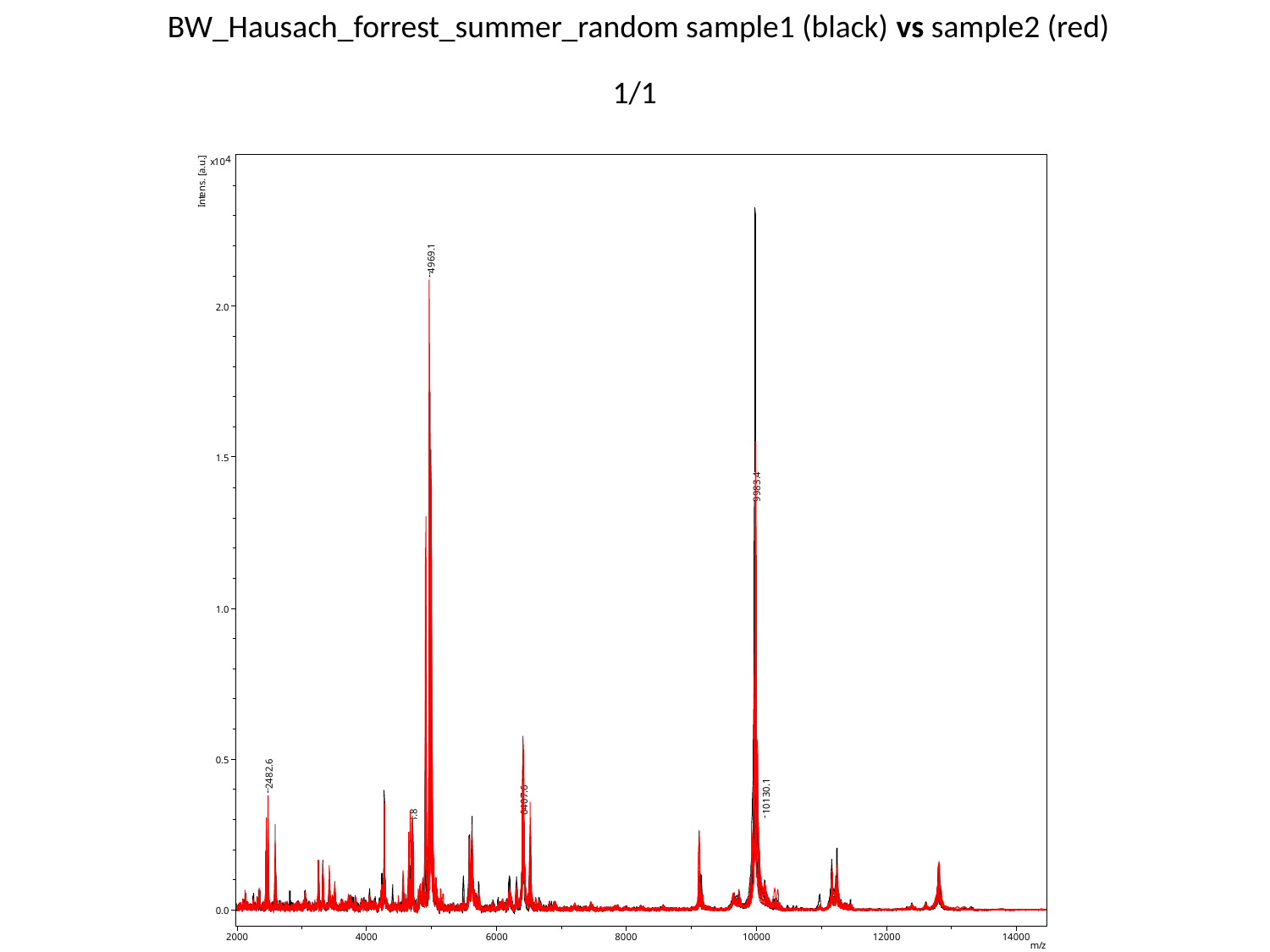

BW_Hausach_forrest_summer_random sample1 (black) vs sample2 (red)
1/1

## Slide 14
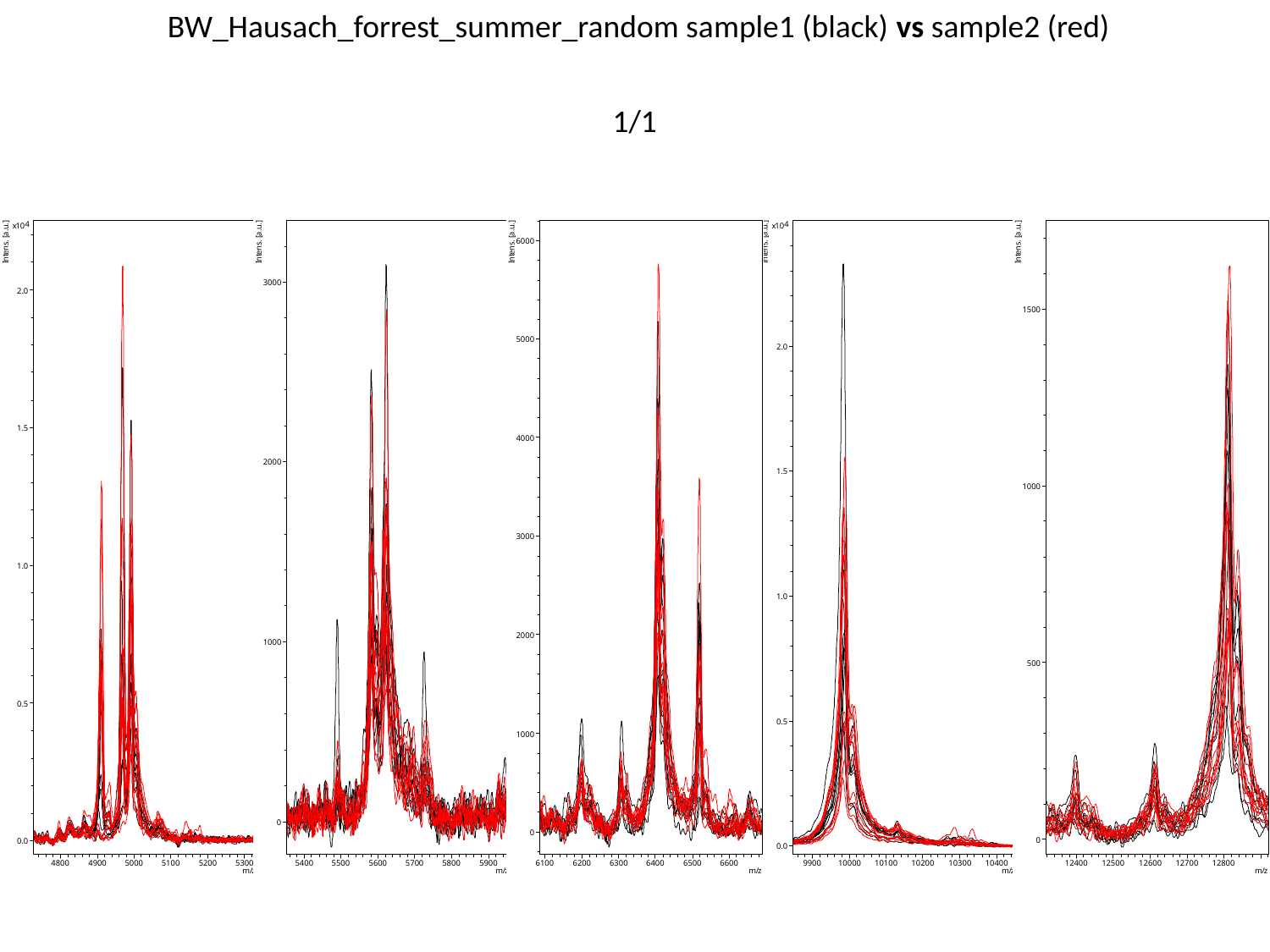

BW_Hausach_forrest_summer_random sample1 (black) vs sample2 (red)
1/1

## Slide 15
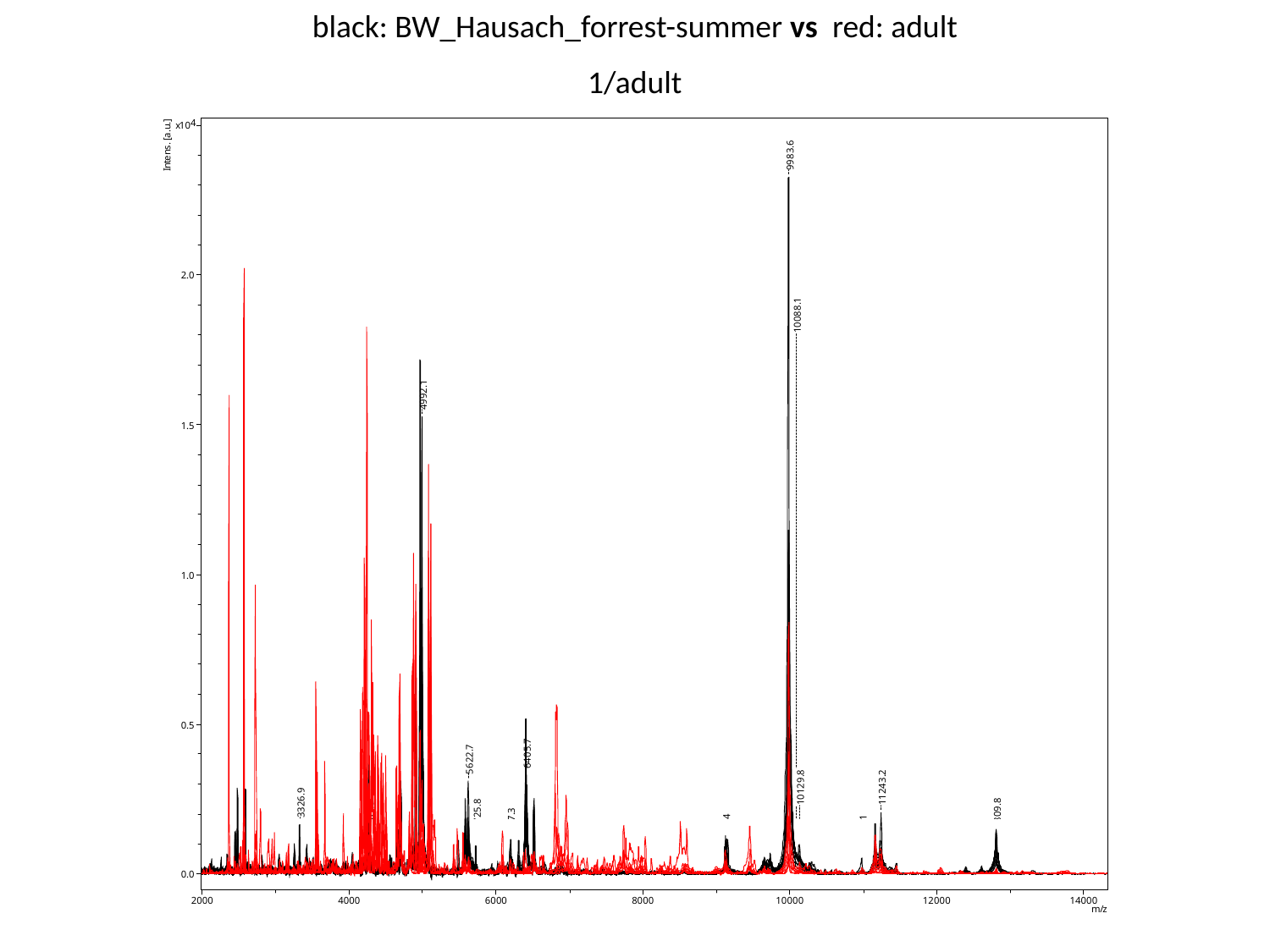

black: BW_Hausach_forrest-summer vs red: adult
1/adult

## Slide 16
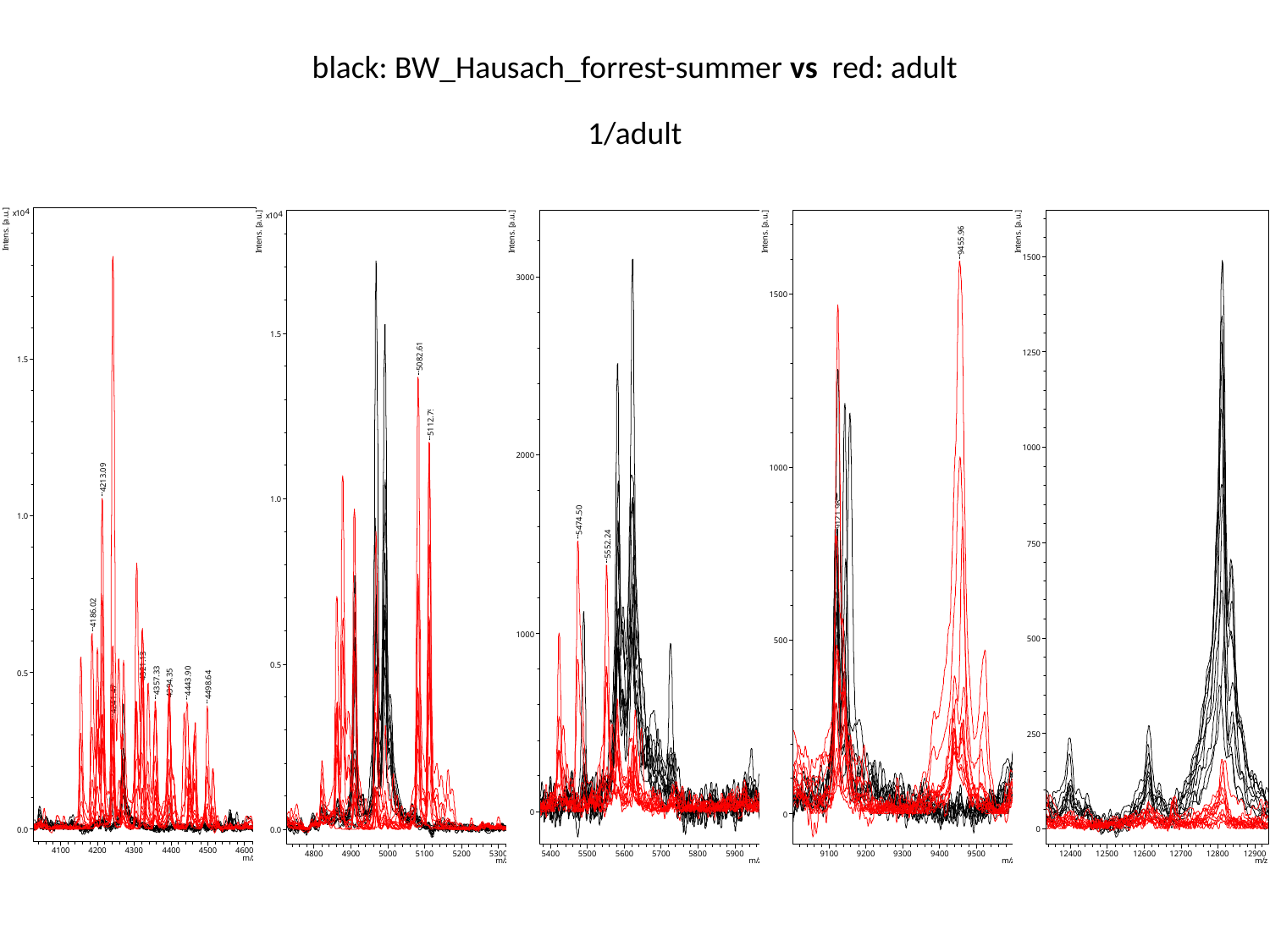

black: BW_Hausach_forrest-summer vs red: adult
1/adult

## Slide 17
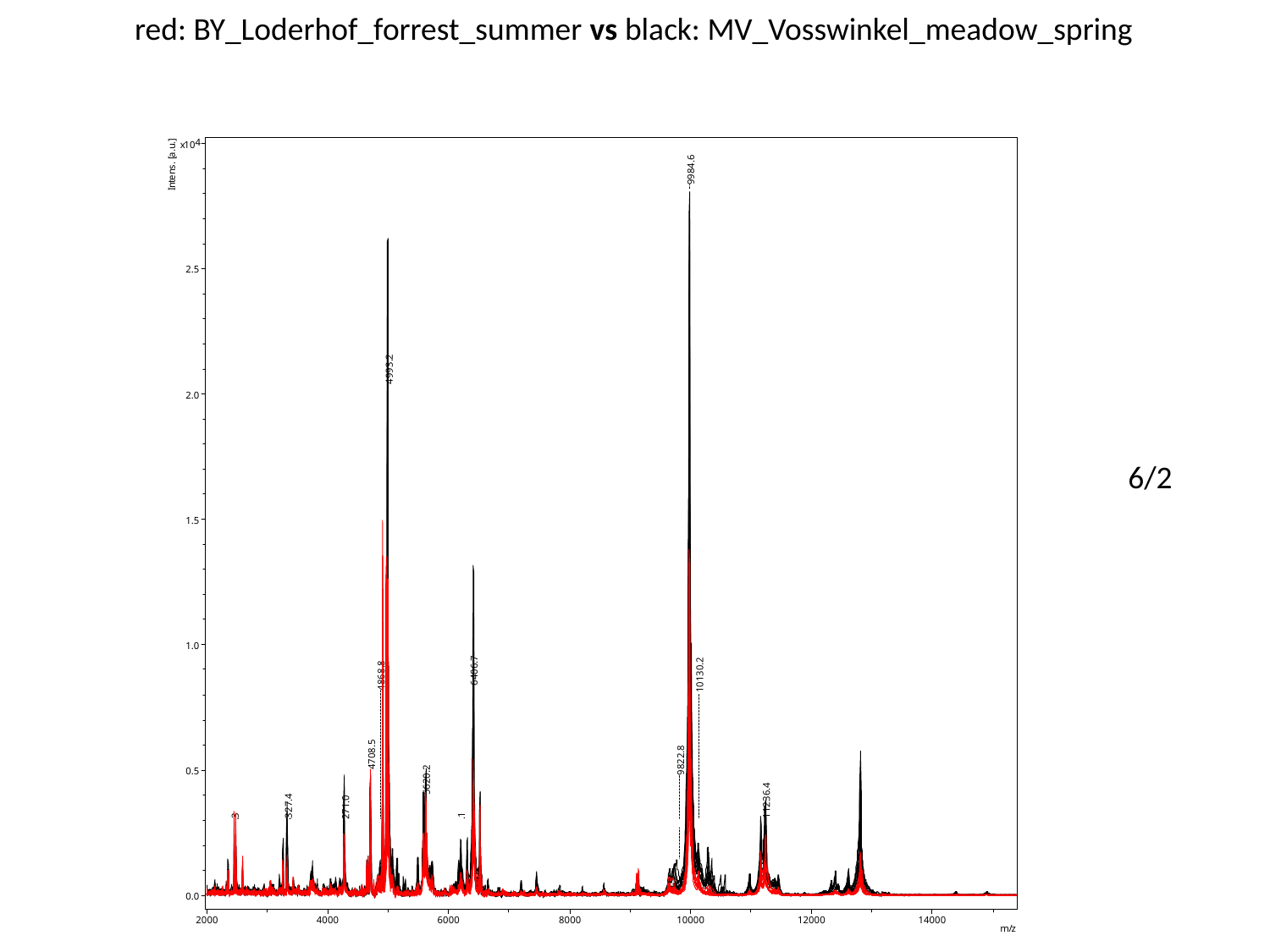

red: BY_Loderhof_forrest_summer vs black: MV_Vosswinkel_meadow_spring
6/2

## Slide 18
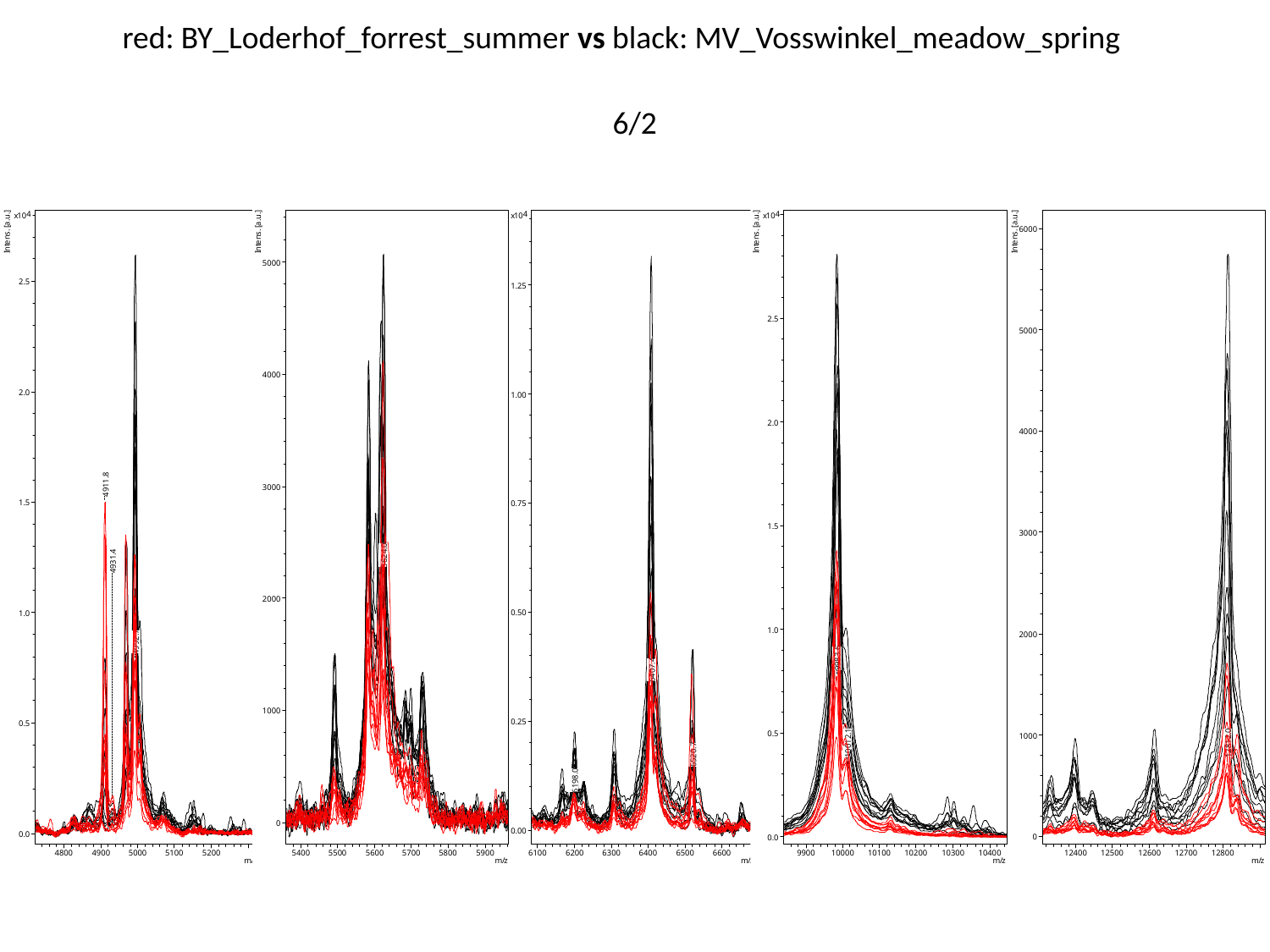

red: BY_Loderhof_forrest_summer vs black: MV_Vosswinkel_meadow_spring
6/2
